# Small nodules (≤ 6 mm in diameter) of multiple primary lung cancers: prevalence and management

**DOI:** 10.1186/s13019-022-02022-2

**Published:** 2022-11-01

**Authors:** Hua Cheng, Wen-hao Li, Xiao-jian Li, Hong-cheng Zhong, Xiao-jin Wang, Yu-jing Lin, Xue-guo Liu, Xiang-wen Wu, Qing-dong Cao

**Affiliations:** 1 Department of Thoracic Surgery, The Fifth Affiliated Hospital of Sun Yat-Sen University, No. 52 Meihua Dong Road, Zhuhai, 519000 Guangdong Province People’s Republic of China; 2 Department of Pathology, The Fifth Affiliated Hospital of Sun Yat-Sen University, Zhuhai, People’s Republic of China; 3 Department of Radiology, The Fifth Affiliated Hospital of Sun Yat-Sen University, Zhuhai, People’s Republic of China

**Keywords:** Multiple primary lung cancer, Non-dominant tumor, Comprehensive histological assessment, Interval growth

## Abstract

**Background:**

Synchronous multiple primary lung cancers associated with small non-dominant nodules are commonly encountered. However, the incidence, follow-up, and treatment of small non-dominant tumors have been but little studied. We explored the prevalence and management of small non-dominant tumors and factors associated with interval growth.

**Methods:**

This observational, consecutive, retrospective single-center study enrolled patients diagnosed with synchronous multiple primary lung cancers and small non-dominant tumors (≤ 6 mm in diameter) who underwent resection of the dominant tumor. The incidence, follow-up, and management of small non-dominant tumors and predictors of nodule growth were analyzed.

**Results:**

There were 88 patients (12% of all lung cancer patients) with pathological diagnoses of synchronous multiple primary lung cancers. A total of 131 (18%) patients were clinically diagnosed with at least one small (≤ 6 mm in diameter) multiple primary lung cancer non-dominant tumor. 94 patients with 125 small-nodule non-dominant tumors clinically diagnosed as multiple primary lung cancers were followed-up for at least 6 months. A total of 29 (29/125, 23.2%) evidenced small pulmonary nodules (≤ 6 mm in diameter) that exhibited interval growth on follow-up computed tomography (CT). On multivariate analysis, a part-solid nodule (compared to a pGGN) (OR 1.23; 95% CI 1.08–1.40) or a solid nodule (compared to a pGGN) (OR 3.50; 95% CI 1.94–6.30) predicted small nodule interval growth.

**Conclusion:**

We found a relatively high incidence of multiple primary lung cancers with small non-dominant tumors exhibiting interval growth on follow-up CT, suggesting that resection of non-dominant tumors at the time of dominant tumor resection, especially when the nodules are part-solid or solid, is the optimal treatment.

**Supplementary Information:**

The online version contains supplementary material available at 10.1186/s13019-022-02022-2.

## Introduction

Lung cancer screening reduces lung cancer mortality in high-risk populations [1, 2]. Small, solitary pulmonary nodules must always be followed-up. The nodule size threshold for surgical resection is 6–8 mm [3]. Current multiple primary lung cancer guidelines emphasize management of the dominant tumor. In the clinic, some multiple primary lung cancer patients exhibit dominant tumors longer than 6–8 mm and non-dominant tumors shorter than 6 mm. No guideline aiding non-dominant tumor selection and treatment is yet available.

Small nodules are commonly encountered during lung cancer screening and can develop into invasive cancers [4]. Nodule size and growth rate determine the nature of the nodule, and its management [5]. However, the use of only nodule dimensions is limited in terms of predicting whether small nodules can develop into advanced lung cancers [4, 5]. In multiple primary lung cancer patients with small non-dominant tumors, it remains unclear whether to resect multiple primary lung cancers and non-dominant tumors at the same time or in separate operations. Here, we explored the prevalence of multiple primary lung cancer with small-nodule non-dominant tumors; the management strategy; and predictors of nodule growth.

## Material and methods

We established a cohort of consecutive patients with non-small cell lung cancer who underwent radical resection from January 2018 to July 2020. Patients with synchronous multiple primary lung cancers and small non-dominant tumors (≤ 6 mm) who underwent dominant tumor resection were included in the study. We reviewed all computed tomography (CT) images. For patients with more than one pulmonary nodule, we also examined the medical records and pathology reports. The largest or most invasive nodule was the dominant tumor and the other nodules possible non-dominant tumors. The resected dominant tumors had been pathologically diagnosed as primary lung cancers. The surgical procedures were based on tumor size, location, CT features, performance status, and pulmonary function. Solid and part-solid dominant tumors were usually resected via lobectomy or segmentectomy. non-dominant tumors were always resected via segmentectomy or wedge resection. For patients exhibiting a poor pulmonary reserve or poor performance status, segmentectomy or wedge resection was selected instead of lobectomy.

Tumor morphology, size, and growth aided the clinical diagnosis and identification of benign and malignant lung nodules. The likelihood of malignancy correlates with both nodule size and growth rate, and any history of prior lung cancer or an extrathoracic malignancy. All multiple primary lung cancer diagnoses were reviewed by our multidisciplinary lung cancer team.

The pathologically diagnostic criterion for multiple primary lung cancers was a comprehensive histological assessment (based on our previous work) [6]. Briefly, we semi-quantitatively evaluated the relative percentages of all histological subtypes (lepidic, acinar, papillary, micropapillary, and solid components) in 10% increments. Paired tumors exhibiting similar histological features were considered to be lung metastases, and those exhibiting different proportions of histological subtypes or features to be multiple primaries. Tumors presenting as adenocarcinomas in situ (AISs), minimally invasive adenocarcinomas (MIAs), or lepidic-predominant adenocarcinomas (LPAs), were considered to be multiple primaries. We performed a targeted gene panel test to distinguish multiple primaries from intra-pulmonary metastases.

The maximum diameters of all nodules were measured with electronic calipers using our institutional picture archiving and communication systems. Nodules were considered to exhibit interval growth if the axial size increased by ≥ 1 mm; this represents almost a volumetric doubling of a nodule ≤ 6 mm. Volumetric analysis was not available given the retrospective nature of the study. The study was approved by the Institutional Review Board and the Ethics Committee of the Fifth Hospital of Sun Yat-sen University and written informed consent was obtained from all patients.

### Statistical analysis

Kolmogorov–Smirnov test and Shapiro–Wilk test were used to test normality for continuous variables. The student t-test or the Mann–Whitney U test was used to compare continuous variables between the two groups and the chi-squared or Fisher exact test was employed to compare categorical variables. To investigate the factors associated with the clinical characteristics of patients and the presence or growth of nodules, a one-way ANOVA was first performed, and variables with significant differences (*P* < 0.1) were selected and then included in a multifactorial logistic regression analysis. SPSS software (IBM SPSS Statistics 25.0, USA) was used to analyze and compare the data. A two-tailed *P* value < 0.05 was considered significant.

## Results

From January 2018 to July 2020, 735 patients underwent resection of primary lung cancers in our department. CT revealed that 257 (35%) had more than one nodule. Eighty-eight (12%) patients were pathologically diagnosed with synchronous multiple primary lung cancers.

### Factors associated with multiple primary lung cancers

On multivariate regression, sex and nodule density were associated with multiple primary lung cancers (Table 1). Such cancers were more common in women than men (odds ratio [OR] 2.49; 95% confidence interval [CI] 1.20–5.14). Multiple pure ground-glass nodules (pGGNs) were more likely to be multiple primary lung cancers than were solid nodules (OR 1.36; 95% CI 1.13–1.31).

**Table 1:** Multivariate analysis of factors associated with multiple primary lung cancer

Predictors	OR (95% CI)	*P* value
Female versus male	2.49 (1.20, 5.14)	0.013
Upper lobe versus middle or lower lobe	1.36 (0.97,1.91)	0.072
pGGN VS solid	1.36 (1.13, 1.31)	0.012

### The prevalence of small pulmonary nodules

A total of 177 (24%) patients had small nodules identified on at least one CT examination. Of these, 131 (18%) had a clinical diagnosis of multiple primary lung cancer non-dominant tumors (≤ 6 mm) and, of these, 43 (5%) underwent surgical resection, including 38 at the time of dominant tumor resection and 5 again after follow-up CT revealed significant nodule growth. There were no perioperative deaths (30 or 90 days). After surgery, a total of 35 (83%) small nodules in 32 (4.3%) patients were found to be pathologically diagnosed as multiple primary lung cancer, including 4 small nodules as ais, 9 small nodules as mia, and 22 small nodules as invasive adenocarcinoma. In addition, two patients with a total of three small nodes were pathologically diagnosed as metastatic carcinoma, and three patients with a total of five small nodes were pathologically diagnosed as non-malignant nodes. The malignancy of all resected small nodules was 88%.

### Development of lung cancer from small pulmonary nodules

**Fig. 1:**
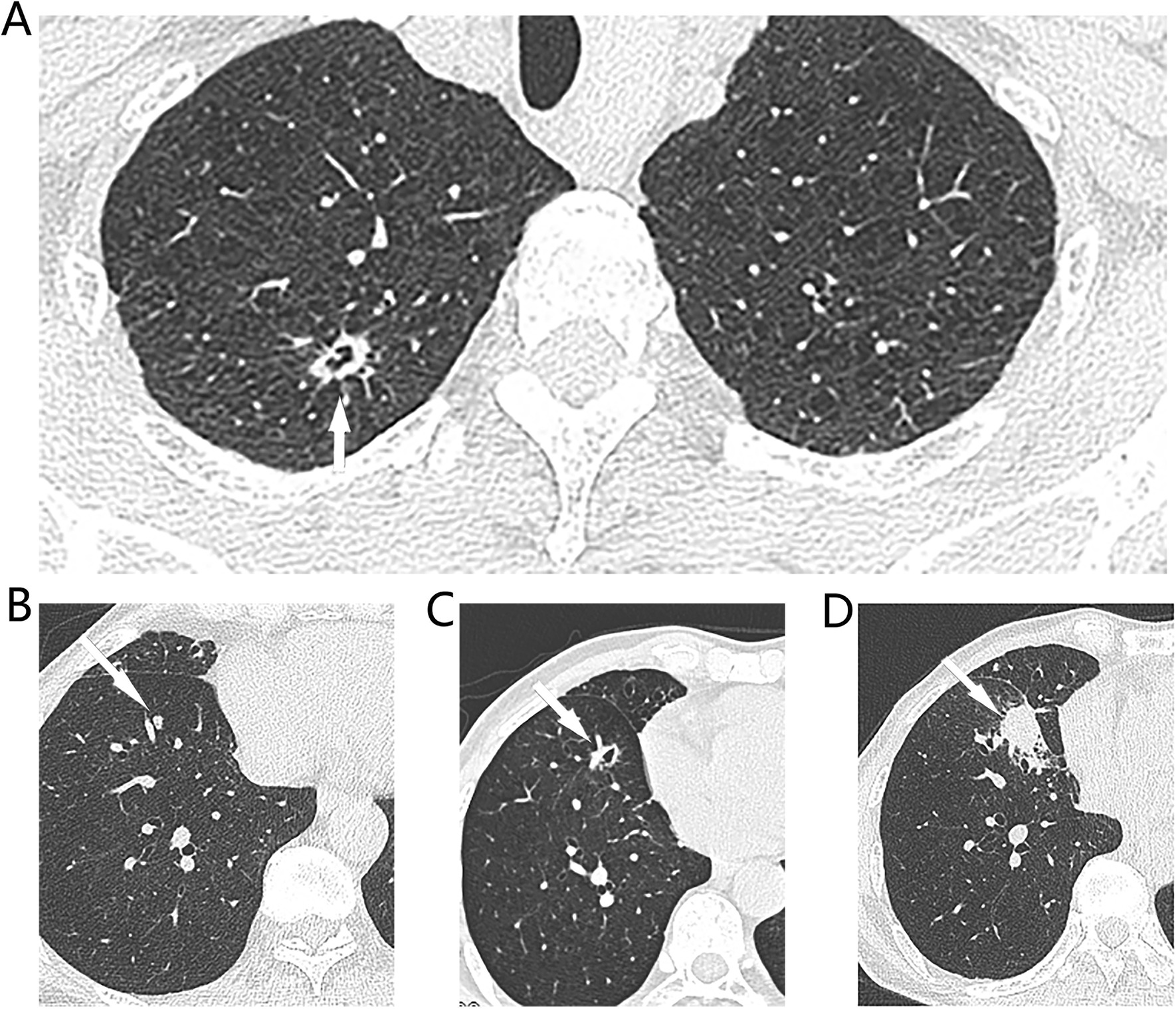
Typical CT images of synchronous multiple primary lung cancers. **A** On preoperative CT of a 67-year-old male patient shown a 13*8 mm cystic nodule (white arrow) in the right upper lobe, and **B** a 5 mm solid nodule (white arrow) in the S8 segment of right lower lobe. Patient received right upper lobectomy and systemic lymph node dissection in July 2019 and confirmed as adenocarcinomas (80% acinar adenocarcinoma, 10% solid adenocarcinoma) on surgical pathology, pT1bN0M0, IA2. The small solid indetermined nodule of the S8 segment of right lower lobe was followed up. **C** 5 months later, the nodule had grown up to a 10 mm cystic nodule. **D** 14 months later, this nodule had grown up to a 24*20 mm solid nodule and with multiple N2 lymph node enlargement, CT guided core needle biopsy shown acinar predominant adenocarninoma. This patient was diagnosed with synchronous multiple primary lung cancers. Because of multiple N2 lymph node metastasis and no driver gene mutation, he received Anti-PD-1 Therapy plus Chemotherapy

Overall, 94 patients with 125 non-dominant tumors suspected of being multiple primary lung cancers were followed-up for at least 6 months. Of these, 17 underwent follow-up CT for ≤ 365 days and 77 follow-up CT for > 365 days. The median follow-up time was 530 (416–585) days. Of these patients, 19 had 29 (29/125, 23.2%) small pulmonary nodules (≤ 6 mm) evidencing interval growth on follow-up CT (Additional file 1: Fig. S1, Additional file 2: Fig. S2) and 4 with 5 nodules underwent surgical resection; 3 were pathologically confirmed to have three multiple primary lung cancers; 1 had two pulmonary nodules that were metastases from colon cancer; and 1 was pathologically diagnosed with multiple primary lung cancer via CT-guided core needle biopsy (several N2 lymph nodes were cancer-positive). The other 14 patients were followed-up (Table 2). The medium volume doubling time was 347 (233–487.5) days. Figure 1 shows a patient with an multiple primary lung cancer and a 5-mm solid nodule in the right lower lobe that exhibited interval growth from 5 to 24 mm over 14 months, accompanied by multiple N2 mediastinal lymph node metastases. CT-guided core needle biopsy of the right lower lobe revealed an invasive adenocarcinoma. Comprehensive histological assessment diagnosed multiple primary lung cancers.

**Table 2:** Characteristics of non-dominant small nodules with interval growth

Patient ID	Nodule ID	Gender	Number of tumors	Characteristics of dominant tumor			Characteristics of non-dominant tumor with interval growth					
				Maximum diameter(mm)	CT characteristic	Histology	Baseline Diameter (mm)	Diameter of latest measurement (mm)	Volume double time (Day)	CT characteristic	Surgical resection	Histology
1	1	F	3	14*13	PSN	Ad	4	12	232	pGGN	Yes	Ad
2	2	F	5	15*13	PSN	Ad	3	14	157	SN	Yes	Ad
2	3						5	6	2012	pGGN	No	NA
3	4	M	2	10*8	PSN	Ad	4.5	10.5	295	PSN	Yes	Ad
4	5	M	2	20*18	PSN	Ad	4	5.5	353	PSN	No	NA
5	6	M	4	16*10	PSN	Ad	5.5	8	451	pGGN	No	NA
6	7	M	2	12*10	PSN	Ad	5	24	63	SN	No (core needle biopsy)	Ad
7	8	F	3	14*10	GGN	Ad	3.2	4.3	329	SN	No	NA
8	9	M	2	34*32	SN	Ad	4	8	186	SN	No	NA
9	10	F	3	14*14	GGN	Ad	2	10	84	SN	Yes	M
9	11						2	10	84	SN	Yes	M
10	12	F	5	41*32	PSN	Ad	4	8	280	pGGN	No	NA
10	13						3.0	5.6	379	PSN	No	NA
11	14	F	4	15*7	PSN	Ad	4.5	5.5	950	PSN	No	NA
12	15	F	2	12*11	pGGN	Ad	5	8	227	PSN	No	NA
12	16						3.8	6.8	234	SN	No	NA
13	17	F	7	7*6	PSN	Ad	2.7	4.8	450	PSN	No	NA
13	18						4.11	5.21	1399	SN	No	NA
14	19	F	10	29*15	PSN	Ad	3.4	5	524	PSN	No	NA
15	20	M	5	10*10	SN	Ad	4	6	1554	pGGN	No	NA
15	21						2.5	4	1554	pGGN	No	NA
16	22	F	2	11*5	PSN	Ad	2	6	376	SN	No	NA
17	23	M	3	22*17	PSN	Ad	4.4	7.2	354	SN	No	NA
18	24	F	3	9*8	PSN	Ad	5.5	8.9	347	PSN	No	NA
19	25	F	6	16*10	SN	Ad	4.2	5.8	906	SN	No	NA
19	26						3.8	6.3	292	SN	No	NA
19	27						3.9	6.6	292	SN	No	NA
19	28						4.5	8.1	292	SN	No	NA
19	29						3.6	5.9	396	SN	No	NA

M, Male; F, Female; PSN, Part-solid nodule; SN, Solid nodule; pGGN, Pure ground glass nodule; Ad, Adenocarcinoma; M, Metastasis

### Factors associated with pulmonary nodule growth

On univariate analysis, nodule density on CT [part-solid vs. pGGN (OR 4.18; 95% CI 1.12–14.94), solid vs. pGGN (OR 11; 95% CI 3.175–38.74)]; and a history of cancer (OR 5.5; 95% CI 1.24–24) were associated with nodule growth.

On multivariate analysis, a part-solid nodule versus a pGGN (OR 1.23; 95% CI 1.08–1.40); a solid nodule versus a pGGN (OR 3.50; 95% CI 1.94–6.30) were predictors of small nodules exhibiting interval growth (Table 3).

**Table 3:** Univariate and multivariate analyses for factors associated with interval growth of small non-dominant tumors

Independent variables	Univariate predictors		Multivariate predictors	
	OR (95%CI)	*P* value	OR (95%CI)	*P* value
Age	0.43 (0.092, 2.024)	0.14		
Sex				
Male	Reference			
Female	0.59 (0.21, 1.63)	0.41		
Smoke				
None smoker	Reference			
Current smoker	3.57 (1.09, 11.6)	0.03	1.06 (0.45, 2.48)	0.89
Former smoker	1.6 (0.53, 5.19)	0.37		
CT density				
Pure GGN	Reference			
Part solid	4.18 (1.12, 14.94)	0.03	1.23 (1.08, 1.40)	0.029
Solid	14.7 (4.74, 46.09)	< 0.001	3.50 (1.94, 6.30)	< 0.001
Nodule margin				
Regular	Reference			
Irregular	2.2 (0.37, 13)	0.19		
Spiculated	2.73 (0.76, 9.8)	0.06	1.08 (0.98, 1.19)	0.11
Lobulated	0.93 (0.17, 4.8)	0.46		
Nodule location				
Upper lobe				
Middle or lower lobe	0.32 (0.48, 1.26)	0.34		
History of cancer	5.5 (1.24, 24)	0.014	1.15 (0.81, 1.6)	0.072

## Discussion

Given the widespread use of high-resolution chest imaging systems and lung cancer screening programs, patients with multiple primary lung cancers are growing in numbers worldwide [7]. Any solitary small nodule ≤ 6–8 mm should be followed-up in terms of growth [8]. The clinical management of such patients varies by the risk of malignancy and (to some extent) the patient and the referring physician. For patients with multiple nodules, the Fleischner Society and National Cancer Comprehensive Network guidelines recommend that multiple primary lung cancer management should be based on the dominant tumor [3]; no recommendations for the selection and treatment of non-dominant tumors are made. When a patient exhibits a small nodule that raises a suspicion of an non-dominant tumor, should we resect this along with the dominant tumor or during follow-up? No consensus has yet emerged. In this study, 4.3% of patients exhibited small, pathologically diagnosed multiple primary lung cancers, 88% of resected small suspicious nodules were malignant and there was no operative mortality. Contemporaneous resection of the dominant tumor and a small suspicious nodule eradicates the tumor early and avoids the risk of advanced disease. Some patients may be unwilling to undergo a second operation or may be unfit. Also, resection of small suspected non-dominant tumors allows of precise diagnosis and staging, usefully guiding adjuvant therapy. In this study, partially solid and solid nodules predicted nodule growth. Clinically suspicious multiple primary lung cancers with small non-dominant tumors should undergo limited resection at the time of dominant tumor removal if the possible non-dominant tumors are ipsilateral to the dominant tumor and are part-solid or solid.

The probability of a malignant, small pulmonary nodule in those undergoing lung cancer screening is low and correlates with nodule size. In the NELSON trial, the lung cancer probability was 0.4% when the nodule diameter was < 5 mm, and 1.3% when the diameter was 5–10 mm [9]. In the National Lung Screening Trial (NLST), the lung cancer probability was 0.3% when the nodule diameter was 4–6 mm [10]. Munden et al. [4] analyzed the NLST data and found that 42% of all participants had at least one CT-visible micronodule (less than 4 mm). In 13 cases, micronodules developed into lung cancer; the rate was thus 0.11% (13 of 11,326) of all subjects with micronodules.

However, the implications of small nodules in oncology patients are much more serious; it is not always practical to wait for a long time. Khokhar et al. [11] reported that 42% of pulmonary nodules were malignant among patients with prior extrapulmonary cancer histories. Hanamiya et al. [12] evaluated 308 patients with extrapulmonary carcinomas or sarcomas. At least one non-calcified pulmonary nodule was detected in 75% (233/308); 7% of all small nodules (< 5 mm) were malignant, as were 4% of nodules of 5–10 mm and 15% of those > 10 mm. In the present study, 35 of 42 (83%) resected suspected non-dominant tumors were multiple primary lung cancers. Of 125 small nodules that were followed-up, 29 (23.2%) grew.

Resection of small possible non-dominant tumors raises the concern of overdiagnosis; such nodules may not cause any harm to the patient if they are not removed. In fact, the extent of overdiagnosis on lung cancer screening has been grossly exaggerated. Patz et al. [13] reported that over 18% all lung cancers detected by low-dose (LD) CT during the NLST were overdiagnoses. However, this is likely an overestimate because of the potential lead-time bias associated with the LD CT arm. After extended follow up of NLST, the median follow-up times were 11.3 years for cancer incidence and 12.3 years for mortality. The lung cancer incidence in the LD CT arm was the same as that in the chest X-ray arm; the overdiagnosis rate was 3.1% [14]. Some overdiagnosis is inevitable when dealing with small non-dominant tumors. Resection of small suspicious non-dominant tumors avoids a second operation and the risk of advanced disease, at a cost of a small overdiagnosis rate.

Our work has several limitations. First, it was retrospective in nature; some recording bias may be inevitable. The criteria used to select small nodules for resection may differ among surgical teams. The sample size was relatively small and the risk of nodule growth may have been underestimated. The follow-up period may have been too short to detect progression of slow-growing cancers, particularly GGNs. However, we are the first to describe a relatively high incidence of multiple primary lung cancers with small non-dominant tumors, no operative mortality, and predictors of interval growth.

## Conclusion

Multiple primary lung cancers with small non-dominant tumors are rather common. Such non-dominant tumors can grow or trigger advanced disease. Contemporaneous resection of clinically suspicious multiple primary lung cancers and small non-dominant tumors is recommended if the non-dominant tumors are located ipsilateral to the dominant tumors, and when the nodules are part-solid or solid.

## Supplementary Information

**Additional file 1**. Number 1–17 of 29 small pulmonary nodules (≤ 6 mm) with interval growth demonstrated on follow-up CT.

**Additional file 2**. Number 18–29 of 29 small pulmonary nodules (≤ 6 mm) with interval growth demonstrated on follow-up CT.

## Data Availability

The data that support the findings of this study are available on request from the corresponding author. The data are not publicly available due to privacy or ethical restrictions.

## Abbreviations

SN: Solid nodule
PSN: Part-solid nodule
pGGN: Pure ground glass nodule
AIS: Adenocarcinoma in situ
MIA: Minimally invasive adenocarcinoma
Ad: Adenocarcinoma
M: Metastasis
