# Supplementary figures and images for: Small nodules (≤ 6 mm in diameter) of multiple primary lung cancers: prevalence and management
Source: J Cardiothorac Surg. 2022 Nov 1;17:278. doi: 10.1186/s13019-022-02022-2 (PMC9628062; doi:10.1186/s13019-022-02022-2)

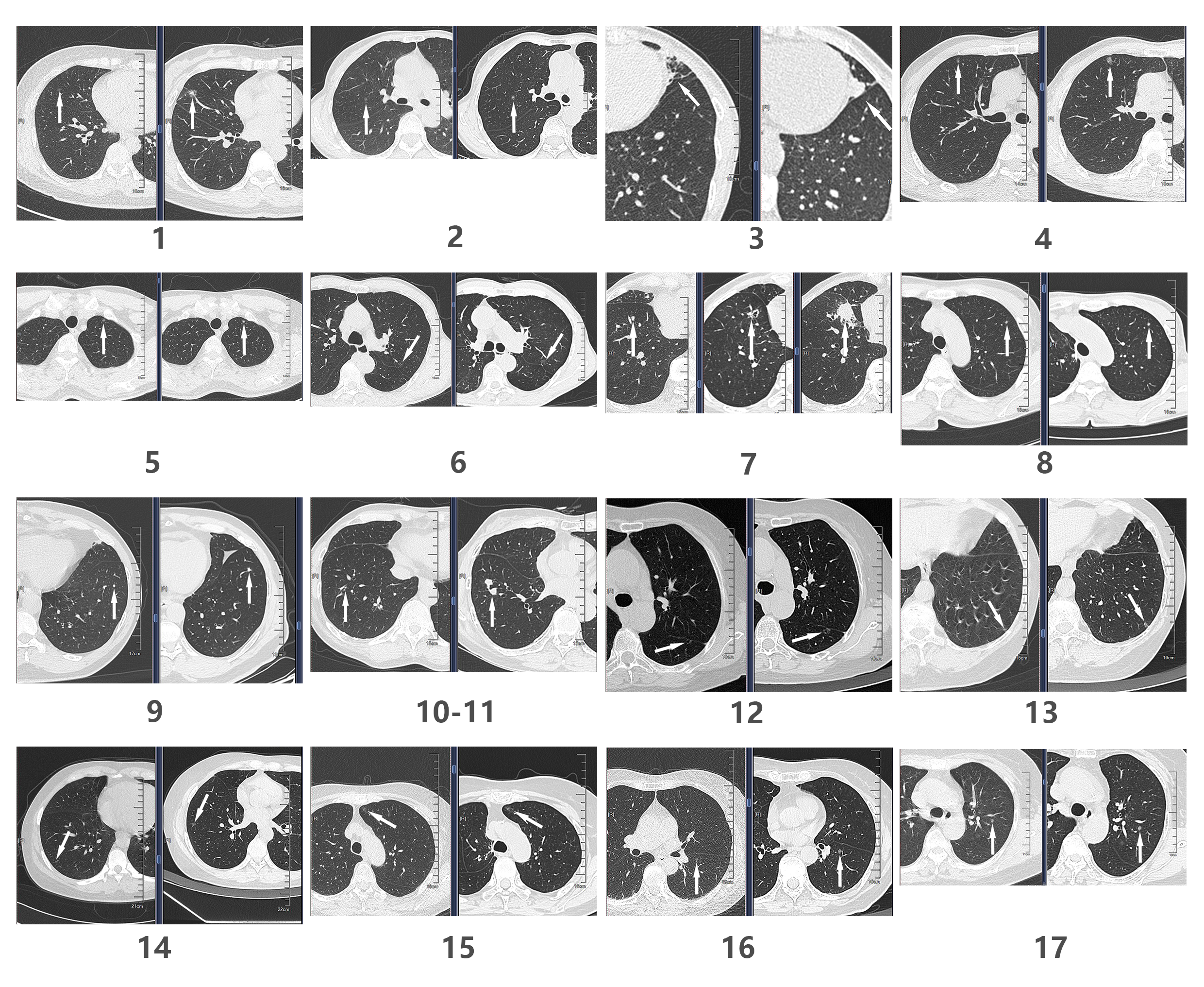

Supplement: Supplementary file 1 — Additional file 1. Number 1–17 of 29 small pulmonary nodules (≤ 6 mm) with interval growth demonstrated on follow-up CT. [file 13019_2022_2022_MOESM1_ESM.tif]

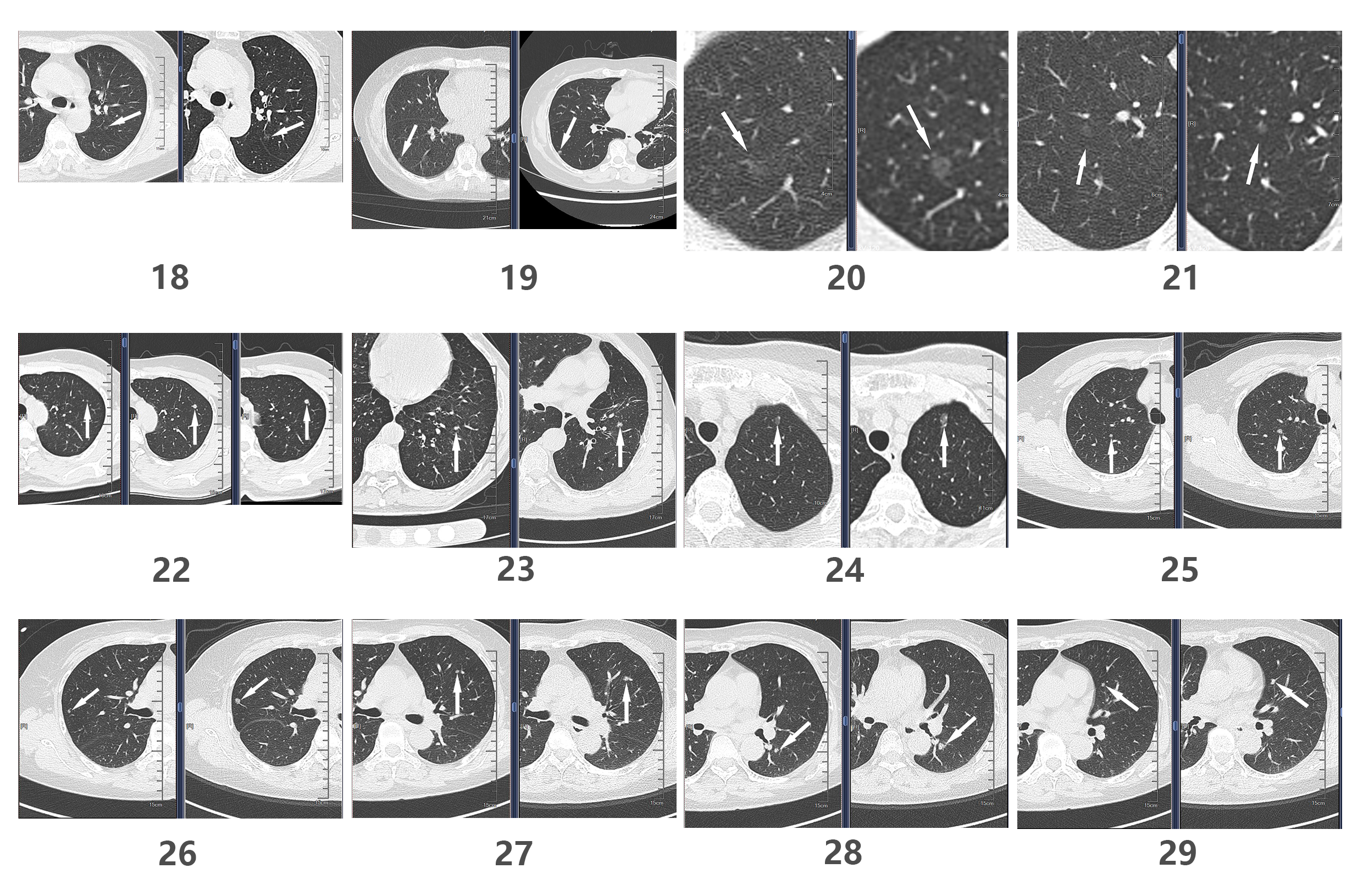

Supplement: Supplementary file 2 — Additional file 2. Number 18–29 of 29 small pulmonary nodules (≤ 6 mm) with interval growth demonstrated on follow-up CT. [file 13019_2022_2022_MOESM2_ESM.tif]
